# Estimating the risk of declining funding for malaria in Ghana: the case for continued investment in the malaria response

**DOI:** 10.1186/s12936-020-03267-9

**Published:** 2020-06-01

**Authors:** Rima Shretta, Sheetal P. Silal, Keziah Malm, Wahjib Mohammed, Joel Narh, Danielle Piccinini, Kathryn Bertram, Jessica Rockwood, Matt Lynch

**Affiliations:** 1 Johns Hopkins Center for Communication Programs, 111 Market Pl Ste 310, Baltimore, MD 21202 USA; 2 Centre for Tropical Medicine and Global Health, Nuffield Department of Medicine, University of Oxford, Oxford, UK; 3 Modelling and Simulation Hub, Africa, Department of Statistical Sciences, University of Cape Town, Rondebosch, Cape Town, 7700 South Africa; 4 Ghana National Malaria Control Programme, Ghana Health Service, Accra, Ghana; 5 International Public Health Associates, Kensington, MD USA

**Keywords:** Malaria, Ghana, Investment case, Costs, Benefits, Economic, Funding, Financing

## Abstract

**Background:**

Ghana has made impressive progress against malaria, decreasing mortality and morbidity by over 50% between 2005 and 2015. These gains have been facilitated in part, due to increased financial commitment from government and donors. Total resources for malaria increased from less than USD 25 million in 2006 to over USD 100 million in 2011. However, the country still faces a high burden of disease and is at risk of declining external financing due to its strong economic growth and the consequential donor requirements for increased government contributions. The resulting financial gap will need to be met domestically. The purpose of this study was to provide economic evidence of the potential risks of withdrawing financing to shape an advocacy strategy for resource mobilization.

**Methods:**

A compartmental transmission model was developed to estimate the impact of a range of malaria interventions on the transmission of *Plasmodium falciparum* malaria between 2018 and 2030. The model projected scenarios of common interventions that allowed the attainment of elimination and those that predicted transmission if interventions were withheld. The outputs of this model were used to generate costs and economic benefits of each option.

**Results:**

Elimination was predicted using the package of interventions outlined in the national strategy, particularly increased net usage and improved case management. Malaria elimination in Ghana is predicted to cost USD 961 million between 2020 and 2029. Compared to the baseline, elimination is estimated to prevent 85.5 million cases, save 4468 lives, and avert USD 2.2 billion in health system expenditures. The economic gain was estimated at USD 32 billion in reduced health system expenditure, increased household prosperity and productivity gains. Through malaria elimination, Ghana can expect to see a 32-fold return on their investment. Reducing interventions, predicted an additional 38.2 clinical cases, 2500 deaths and additional economic losses of USD 14.1 billion.

**Conclusions:**

Malaria elimination provides robust epidemiological and economic benefits, however, sustained financing is need to accelerate the gains in Ghana. Although government financing has increased in the past decade, the amount is less than 25% of the total malaria financing. The evidence generated by this study can be used to develop a robust domestic strategy to overcome the financial barriers to achieving malaria elimination in Ghana.

## Background

Ghana has made impressive progress in its fight against malaria. Malaria cases and deaths have decreased by over 50% and 65%, respectively, between 2005 and 2015 [1]. Nevertheless, malaria remains a major public health burden in Ghana accounting for 30% of outpatient attendances and 23% inpatient admissions. Malaria is endemic and perennial in all parts of Ghana with seasonal variations more pronounced in the north. *Plasmodium falciparum* accounts for over 95% of malaria infections. The entire population is at risk of malaria infection however, transmission is markedly less intense in large urban centers compared to rural areas [2].

The current strategy is based on the National Malaria Control Strategic Plan (NSP) for 2014–2020, which was finalized in August 2014 by the National Malaria Control Programme (NMCP) [3]. The scope of the strategic plan was to consolidate the gains and accelerate malaria control in the high transmission areas to further reduce malaria burden and move towards establishing lower-transmission areas in Ghana by the end of 2020. The plan calls for reducing the malaria morbidity and mortality burden by 75% by the year 2020 compared to 2012.

The gains experienced in the past decade can be attributed in part due to the increased financing available to scale up effective interventions. In 2016, 73% of households owned at least one insecticide-treated net (ITN) with 52% of children having slept under a net the previous night. 78% of pregnant women received two or more doses of intermittent preventive treatment for pregnancy (IPTp) during their last pregnancy, the highest rate in sub-Saharan Africa. In light of the inter-regional and urban/rural difference in malaria burden, efforts are being made to adapt interventions based on the respective needs of different localities [4].

Since 2003, Ghana has benefited from a succession of grants from Global Fund for AIDS, Tuberculosis and Malaria (Global Fund) [5] with disbursements of USD 408 million. The country currently has two active grants totaling USD 109 million (2018–2020). Several other external donors have provided financial support to the malaria programme including the United States Presidents Malaria Initiative (PMI, the U.K. Department for International Development (DFID), the United Nations Children’s Fund (UNICEF), the World Bank, and the governments of Japan, China, and Cuba [1]. Nevertheless, the country still faces a financing gap estimated at 187 million for 2019–2020 by the Ghana National Strategic Plan for malaria (NSP) [3].

Government contributions made up 38% of the total financing for malaria in 2018 [5]. Curative services for malaria are covered by the National Health Insurance Scheme (NHIS) benefit package which is financed by a mix of funding from earmarked taxes and premiums paid by members. However, collection rates are a challenge and, therefore, revenue received from capitation does not offset any significant portion of the expenditure.

The current level of financial support the country receives from external partners is unlikely to be sustained in the longer term. Between 2002 and 2016, Ghana experienced a five-fold increase in GDP per capita from USD 309 to USD 1517 [6–8]. As Ghana’s economy transitions towards middle income, external donor financing for health is expected to decline, with domestic or other sources of financing having to fill the resulting gap. However, experience with other countries points to a significant time-lag between rising national income and increased government health expenditure unless accompanied by effective advocacy backed by evidence outlining the risks associated with funding withdrawal. Historical evidence demonstrates that almost all resurgence events could be attributed, at least in part, to the weakening of malaria control programmes for a variety of reasons, of which resource constraints were the most common [9].

The aim of this analysis was, therefore, to quantify the epidemiological and economic impact of both a fully-funded malaria response that will achieve malaria elimination and that of a partially-funded response that may potentially lead to disease resurgence. The findings of this work can be used to shape the advocacy strategy for mobilizing increased domestic resources for malaria.

## Methods

This investment case projected the financial requirements of the malaria programme to reach malaria elimination by 2030 and values the economic and financial returns of reducing malaria transmission compared to alternative scenarios. To accomplish this, the investment case leveraged multiple methodologies and data sources. The study design incorporated a variety of quantitative methods: numerical and regression techniques to develop a transmission model to predict the epidemiological impact of various interventions used for malaria control and elimination and economic analysis to estimate the cost and economic impact of the interventions nationally and regionally. The economic analysis was informed by the outputs of a transmission model. All monetary figures are expressed in 2018 constant US dollars (USD) [10].

### Epidemiological model framework

A spatially explicit, compartmental, nonlinear, ordinary differential equation model is an extension of previously published models and have been implemented in R and C++ [11, 12]. The model simulated a range of malaria interventions and estimated their impact on the transmission of *P. falciparum* malaria between 2018 and 2030 nationally and in the three ecological zones in Ghana (Coastal, Forest and Savanna). Historical data from 2012 to 2018 was used to parameterize and fit the model. The key transmission features and drivers of transmission in the model included:

- Infection classes dependent on the level of severity of infection.
- Development and loss of immunity against clinical infection.
- Superinfection.
- Subnational climatic variation (seasonality).
- Importation of infection.

More details on the model have been published elsewhere [11–13].

Interventions modelled included:

- Passive Case detection (routine diagnosis and treatment in health facilities and the community).
- Vector Control:
  - Distribution of LLINs.
  - Distribution of LLINsIRS.
- Healh System Strengthening (supportive supervision, training for improved malaria testing and treatment and supply chain management support).
- Social and behavioural change (SBC) for improved health-seeking behaviour.
- Seasonal malaria chemoprevention (SMC).
- Intermittent preventive treatment (IPTp).

Data sources used were:

- Data from the NMCP (monthly incidence and deaths by district from the Health Management Information System (HMIS)).
- WHO World Malaria Reports and Annexes.
- Peer reviewed literature.
- Expert opinion (for assumptions where data were unavailable).

Four scenarios and two reverse scenarios were developed in collaboration with the NMCP:

- *Baseline scenario* existing set of malaria control activities as implemented in 2018 with intervention coverage levels of 2018.
- *Fully funded response (FFR)* this scenario modelled the impact of a fully funded scenario with the targets as outlined in the NSP. It must be noted that the current NSP was developed as a “malaria control” strategy rather than an “elimination” strategy.
- *Better use of nets* added to the “fully funded response” through the use of SBC (social and behavioural change) to increase the usage of LLINs. Given the low usage of nets in Ghana, interventions to increase usage beyond the estimated 41.7% recorded in 2016 [14–16] were added to simulate increased net use. The interventions modelled were a combination of activities of a “hang-up campaign” as well as SBC and IEC and based on a 2014 study in Ghana [17], where LLIN use by children under five years increased to 77.4% in households where some or all LLINs were hung by a campaign volunteer accompanied by SBC/IEC activities in the community, compared to 53.9% in households without these interventions. These interventions increased the odds of a child sleeping under an LLIN approximately 1.5 times when adjusted for other factors that may explain variation in use (adjusted OR: 1.57; 95% CI 1.09, 2.27; p = 0.02). These odds when applied to 41.7% reported usage with 40% protection given usage (a meta-analysis of protective efficacy from use predicted that LLINs had a protective efficacy of 39.8% (IQR 20.2–50.3%) and 28.5%, (IQR 8.8–47.3%) for IRS. Thus for LLINs there is a median effective protection of 16.6%)) result in a median protective efficacy of 24.9% [18].
- *Increase treatment*-*seeking* from 73 to 90% (through SBC) was added to the “better use of nets” scenario.
- *Reverse scenarios* Reduce the amount of funding for the implementation of activities from the 2018 baseline coverage. Where:
  - Reverse 1: cutting out IRS, SMC and LLINs by 50%.
  - Reverse 2: cutting out IRS, SMC (LLINs remain at 2018 levels).

The scenarios were developed in collaboration with the NMCP. Table 1 describes the scenarios in detail.

**Table 1:** Scenarios modelled

No.	Name	Description	Assumptions
1.	Baseline	Existing set of malaria control activities in 2018 Passive testing and treating of positive malaria cases (community and facility-based) Distribution of LLINs with coverage^a^ and usage levels maintained at 2018 levels IRS coverage continued at 2018 levels (6%) Seasonal malaria chemoprophylaxis continued at 2018 levels IPTp continued at 2018 levels (~ 47%) Maintain proportions of participants who receive 1, 2, 3, 4, 5 doses Distribute routine LLINs to participants of IPTp	No cost and service difference between community and facility-based treatment avenues Mass distribution of LLINs every 3 years, in line with data (Coverage 2016-2018: 32%, 9%, 100%) Proportion of participants who take 1,2,3, 4, 5 doses of IPTp remains constant
2.	Fully-funded response (FFR)	Baseline + Test 100% of all suspected cases and treat 100% of positive cases IRS coverage > 80% (to cover Upper East, Upper West, Northern and Brong Ahafo Region (78% of population of the Savanna zone)) Increase IPTp3 to 80% SMC extended to Northern in 2019	Supportive supervision and training to enable better testing and treating (applied annually per PAR) IRS is an annual cost IPTp costs for dosage only (through existing ANC)
3.	Better use of nets	Fully-funded response + SBC (social and behavioural change) to increase the usage of LLINs	Distribution of LLINs every 3 years, *en masse* SBC costs applied to cover 1/3 of the country per year, allowing for full coverage with every mass distribution Costs applied annually at 1/3 coverage per par Impact of SBC: Increase in net use by 50%
4.	Health System Acceleration	Better use of nets + Increase treatment-seeking from 73% to 90% (through increasing SBC)	Increased SBC costs to increase treatment-seeking Costs applied annually per par
5.	Reverse	Cut IRS Cut SMC Cut nets by 50%	
6.	Reverse 2	Cut IRS Cut SMC	

^a^LLIN coverage determined by LLIN usage and effectiveness at reducing transmission

### Economic analysis

Using a societal perspective and cost of illness approach [19], the economic burden of malaria in 2018 was evaluated. Specifically, (i) direct health system costs, (ii) direct household costs, and (iii) indirect costs were estimated. Table 2 illustrates the framework used.

**Table 2:** Framework for estimating the economic burden of malaria in Ghana

Direct health system costs	Direct household costs	Indirect costs
National and subnational expenditures on malaria interventions	Out-of-pocket expenditures for treatment-seeking	Productivity losses among malaria patients and caregivers Value of life years lost due to premature death

### Direct health system costs

To facilitate the gathering of direct cost data, an interview guide and data entry sheet were developed to collect existing costing data, identify gaps, and locate additional data to fill gaps. These interviews were conducted in a semi-structured format with key malaria programme personnel who were familiar with programme spending patterns and records. Data on government and external spending were collated. National health system costs outside of vertical malaria programme expenditures were included as much as possible to obtain the total actual cost to the health system in Ghana. When expenditures were unavailable, budget figures, National Health Accounts (NHA) and secondary sources such as peer-reviewed or grey literature or deduction were used. Costs of treating outpatient and inpatient malaria cases were obtained from the NHA [20, 21].

Individual costs were extracted and aggregated to obtain estimates of the costs of each intervention. The cost of each scenario was estimated using a cost estimation model fed by outputs of the transmission model. The cost of each scenario was then used to obtain the incremental or additional cost of a fully funded response compared to the baseline. All costs were discounted at 7%. The discount rate used was based on the inflation rate and the expert opinion of economists in-country. Additional file 1: Table S1 contains the cost inputs used in the analysis.

### Direct household costs

Malaria exacts a significant financial burden on households. Malaria patients often pay for transportation to access health facilities, diagnostic services, and medicines. In Ghana, although testing and treatment for malaria are free, prepaid or covered by the NHIS, malaria patients still incur out-of-pocket expenditures (OOP) for transport, food and other expenses not covered through the public sector. To estimate direct household costs on malaria, the number of reported OP and IP malaria cases in 2018 was multiplied by the mean OOP spending (separately for OP and IP cases). Data on OOP was obtained from published literature [22, 23].

### Indirect costs

The economic impact of malaria extends beyond the health system. Patients forego income while recovering from malaria, and caregivers looking after ill children and the elderly also lose out on potential earnings. Society also incurs an indirect cost due to premature deaths through losses in lifetime productivity and in the social value people place in living longer, healthier lives.

To evaluate the economic impact of malaria-related morbidity, the foregone income of malaria patients and caregivers was calculated. The gross domestic product (GDP) per capita per day was obtained from 2018 GDP estimates from World Bank Data [7]. The resulting figure was used as a proxy for the average income per capita and multiplied by the duration of OP and IP illness from published literature and the number of reported OP and IP cases. In addition, the effect of reduced productivity from “presenteeism” was calculating by assuming that adults retuning to work would be 50% less productive for an additional 6 days. This assumption was made based on interviews in Ghana.

A full income accounting approach was used to quantify the economic impact of premature death as postulated by the Lancet Commission on health [24]. Assuming 40 years as the average age of malaria-related adult deaths and 2.5 years as the average death amongst children under 5 years, the average remaining life expectancy of males and females was multiplied by the value of each additional life year (VLY). Life expectancy was retrieved from the Central Statistics Service [18]. One VLY was assumed to be 4.2 times the 2018 GDP per capita of Ghana [24].

Cost savings from reduced public and private expenditures on malaria are likely to spur consumer spending and create new businesses thus injecting more money into the local economy. Throughout the process, overall disposable incomes increase, creating more markets for local businesses. These induced responses result in an economic multiplier or “ripple” effect. A 2011 USAID report [25] estimated that income multipliers in West Africa lay between 1.58 and 2.43. An average multiplier of 2 was, therefore, used for the purposes of this analysis.

### Economic benefits estimation

To estimate the benefits of elimination, the averted costs, cases and deaths were calculated. The mortality and morbidity averted from malaria elimination were obtained by subtracting the estimated cases and deaths in the fully funded scenarios from the corresponding outputs of the “business as usual” scenario. Similarly, the excess cases and deaths in the reverse scenarios were calculated by subtracting from the corresponding outputs of the “business as usual” scenario. These health benefits were calculated using the methodology and inputs previously outlined.

Direct costs averted to the health system includes costs associated with diagnosis and treatment costs of IPs and OPs;

- Direct cost averted to the individual households is out-of-pocket (OOP) expenditures for seeking care; and.
- Indirect cost averted to the society due to patients’ lost productivity due to premature death and morbidity and caregivers reduced economic output.

The benefits of investing and not investing in malaria control and elimination were estimated as the sum of the direct cost savings to the health system from reduced use of outpatient and inpatient health services and reduction in cost of delivering malaria control activities; the direct cost savings to households; and the indirect cost savings of reduced morbidity and mortality from malaria calculated above.

The Net Present Value (NPV) was calculated to obtain the present value of the future revenue generated from elimination using standard economic techniques. The purpose was to give a true picture of the financial value of an investment made today whereby savings would be accrued in the future [19]. The timeframe used for calculating the NPV was 11 years and a 7% discount rate was applied as before.

### Return on investment

To calculate the ROI from malaria investments, the NPV of the benefits of reduced transmission were subtracted from the discounted cost of elimination. The resulting figure was divided by the discounted cost of the fully funded response (compared to baseline). The ROI is interpreted as the economic return from every additional dollar spent on malaria above the business as usual scenario.

### Financial gap

Various sources were consulted to estimate past, present, and future financing for malaria. Projected financing was estimated using projected figures from GOG, the Global Fund and PMI. Many of malaria services are covered under the NHIS via the health insurance levy. These estimated resources are included in under “domestic financing” (obtained from the NMCP).

### Sensitivity analysis

A stochastic sensitivity analysis on the epidemiological and cost outputs of the malaria transmission model was performed. The minimum, median, and maximum malaria cases and deaths predicted by the model for each scenario were used to calculate the minimum, median, and maximum costs. Three hundred random samples were drawn, which generated a range of costs. From the range of costs generated, the minimum, maximum, median, mean, and other percentiles are presented.

### Data collection, tools and analysis

A worksheet was developed in Microsoft Excel^®^ to facilitate the organization of cost data. Analysis of the cost data was conducted in Microsoft Excel to estimate the current and future costs of the malaria activities in each scenario. All quantitative data records (no identifying information), were stored in Microsoft Excel spreadsheets on encrypted, password-protected computers. Data was collected in August 2019.

### Ethical approval

Ethical approval for this study was obtained from the Ethical Review Committee (ERC) of the Ghana Health Service prior to data collection (GHS/RDD/ERC Ref No. 1913445).

### Study limitations

A number of known and unknown factors limit the findings of this report. Due to time and resource constraints, the transmission model estimated sub-national malaria transmission based on three climatic zones. Ideally, higher levels of spatial heterogeneity would be modelled to provide to enable subnational estimates of interventions and costs.

The costs of interventions have been estimated based on available data from the NMCP and proxies when data were unavailable. For example, the costs of outpatients, in-patients and health worker salaries were estimated from the National Health Accounts (NHA). Separating out the cost of interventions in integrated systems is challenging and the analysts have relied on country-level partners to arrive at disaggregated costs. This report utilized reported cases from the HMIS and estimated cases and deaths from WHO World Malaria Reports. The wide variation in these two estimates of burden makes it harder to be sure of the resources required to eliminate the disease.

As Ghana progressively moves from a high to a low burden country the impact of active surveillance on both the epidemiology and cost will need to be incorporated. This was not included due to a lack of historical data to enable fitting the model for impact or cost. The savings observed may well be offset by the increased costs of active surveillance required in elimination settings. At the same time, targeting of interventions rather than ubiquitous coverage to the entire country may reduce the costs of elimination and the financing gap. Without subnational estimates of incidence and coverage, targeted interventions are difficult to estimate and cost. Without an informed and complete understanding of the detailed current cartography of malaria risk and prevalence, future projections of the cost of eliminating malaria face an overwhelming uncertainty.

While employee absenteeism was included in the estimates of benefits, the analysis did not include the economic benefits conferred by reductions in school absenteeism and subsequent improvements in cognitive development due to the lack of empirical evidence to enable converting these estimates to wages earned. Other benefits not included were the potential benefits on tourism, the impact of economic development and housing improvements on malaria transmission as well as regional or cross border externalities.

## Results

### Epidemiological impact of partial *vs* full funding (transmission model predictions and projections)

#### Baseline response

**Fig. 1:**
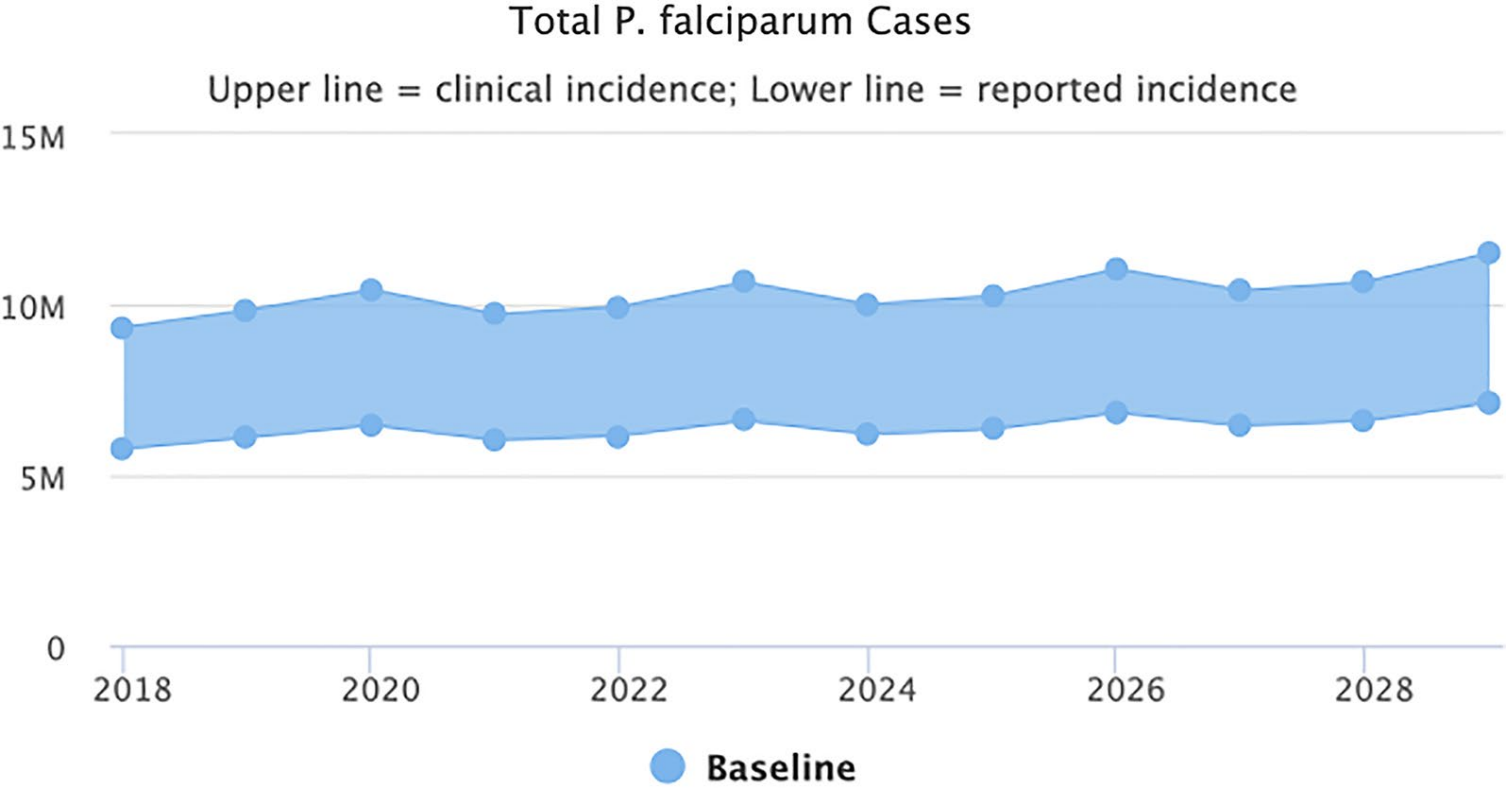
Baseline scenario (business as usual)

Maintaining the interventions (LLIN distribution, IRS, SMC) and health system access and performance at 2018 levels, does not change the transmission intensity. Figure 1 shows that malaria is predicted to continue unabated, with no further decrease expected until 2030 (the end point of the model). The slight upward trend in cases is a reflection of a growing population, rather than increased incidence of malaria. The lower line represents the reported cases using WHO estimates while the upper line represents the estimated clinical cases based on corrections for reporting rate in the public sector. The true number is likely to be somewhere in between.

#### Fully-funded/NSP response

**Fig. 2:**
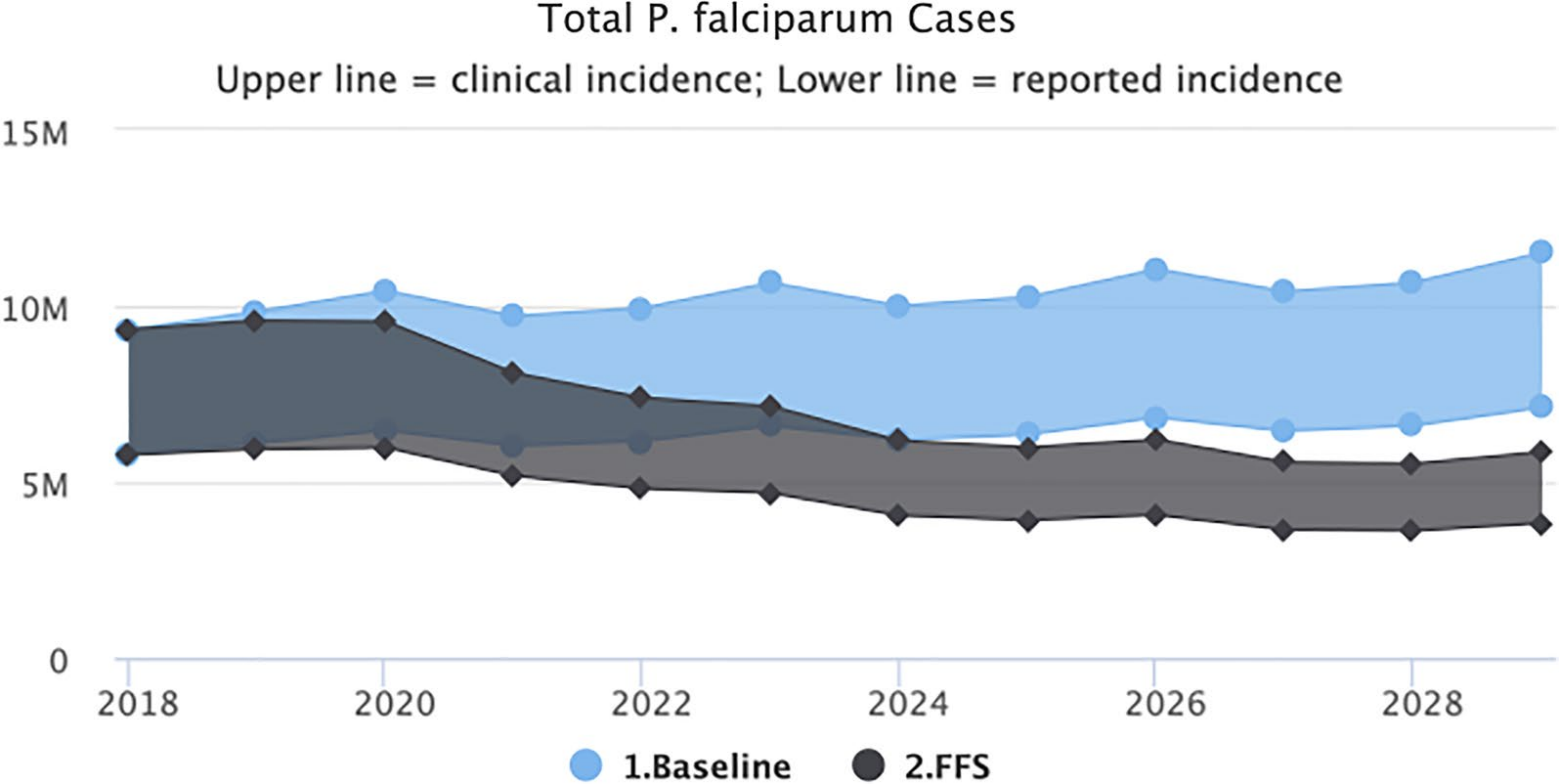
Fully-funded scenario (predicted impact of interventions as outlined in the NSP)

Transmission levels decrease starting in 2020 when the interventions begin. The total number of reported cases however does not fall below 3.5 million annually during the 10-year period (2020–2029), as illustrated in Fig. 2. Compared to the baseline, the fully funded NSP scenario will avert 37.4 million clinical cases, 21.2 million reported cases and 2683 deaths.

#### Better net use

**Fig. 3:**
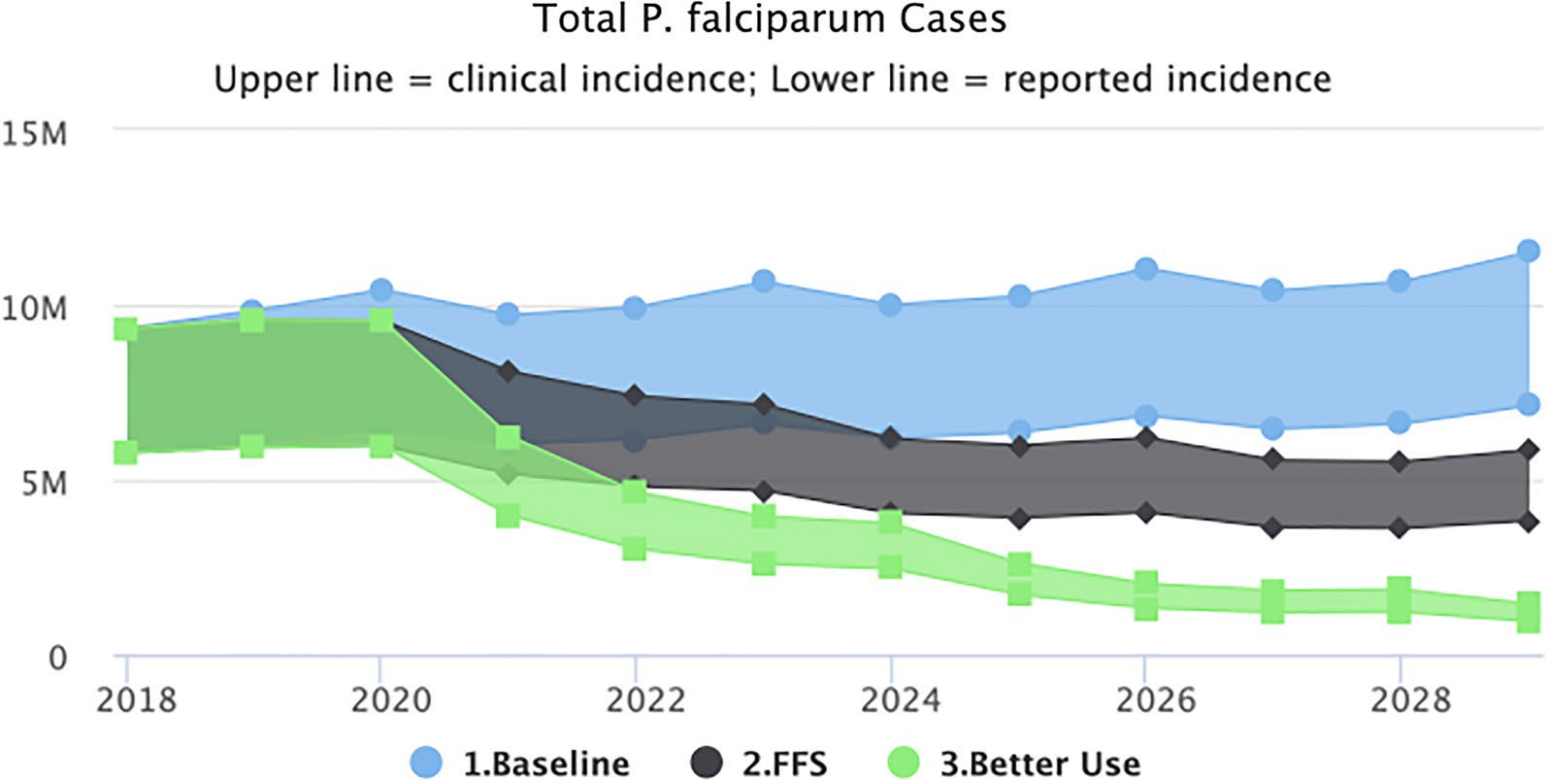
Better net use scenario (predicted impact of interventions as outlined in the NSP plus better use of nets at the household level)

Figure 3 shows that the impact of improved usage results in a considerable predicted decrease in malaria cases at the national level with reported cases falling below 1 million in 2029, however transmission did not fall to elimination levels. These predictions assume that improved usage will be maintained consistently until 2030.

It must be noted that studies have shown that LLIN use varies considerably by urbanization and socio-economic status with low net use being recorded among higher income households in urban areas [25]. Given that the only data available were national averages, it is likely that the impact of net use is overestimated if indeed, use is high amongst the population at risk in rural areas but low amongst those living in low-risk urban areas.

### Health system acceleration

**Fig. 4:**
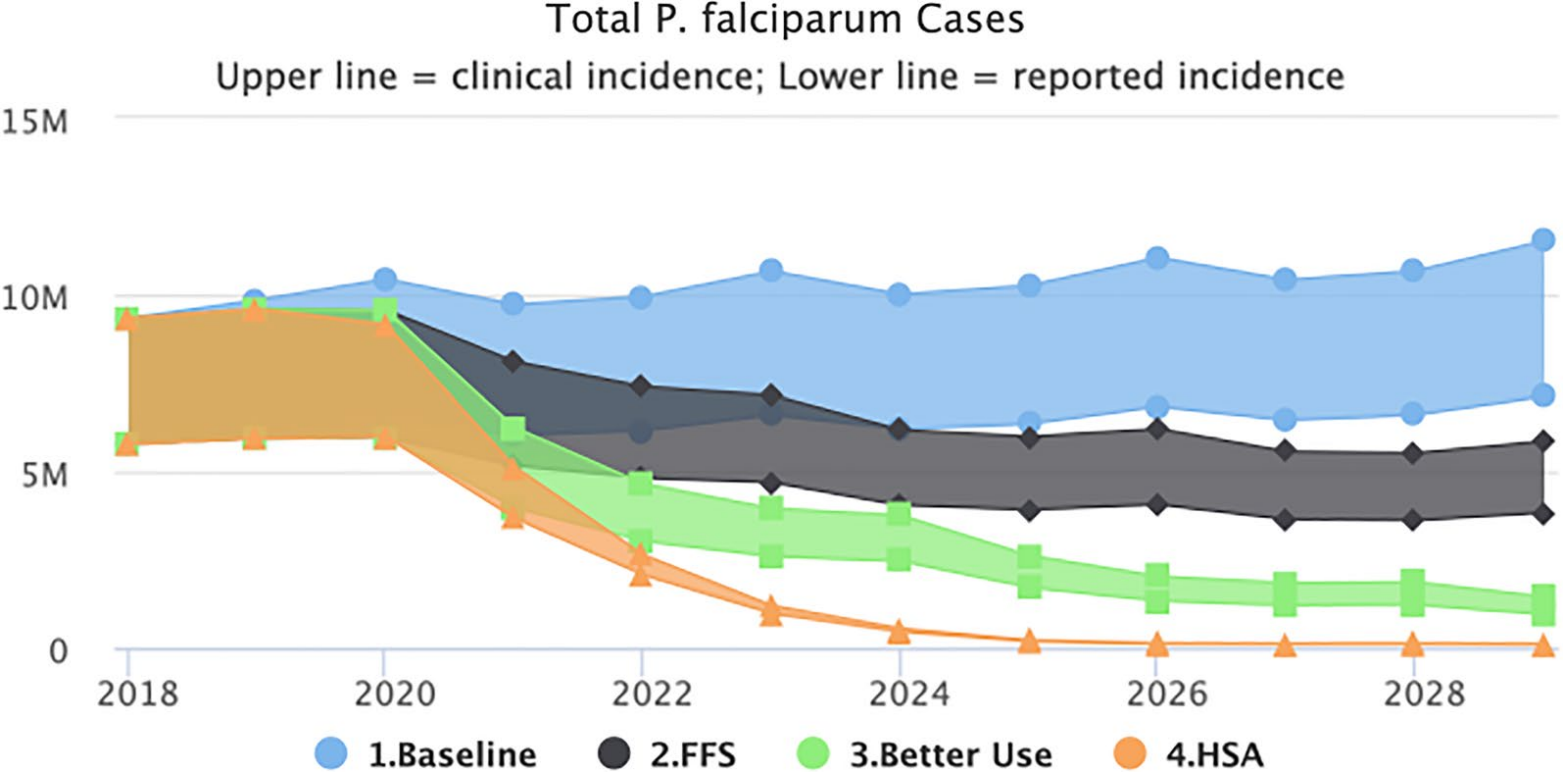
Health system acceleration scenario (predicted impact of interventions as outlined in the NSP plus better use of nets at the household level plus health system strengthening for improved health-seeking behaviours and more effective case management of malaria)

Figure 4 shows the impact of combining better net use with an increase in access to the public health system through SBC and IEC interventions. Modelling this improvement in access as an increase in treatment-seeking behaviour from 73% (reported) to 90%, results in a model prediction of reaching malaria elimination. Compared to the baseline, the HSA scenario will avert 85.6. million clinical cases, 51.3 million reported cases and 4468 deaths, bringing transmission down significantly to near elimination by 2026.

#### Reverse scenario 1

**Fig. 5:**
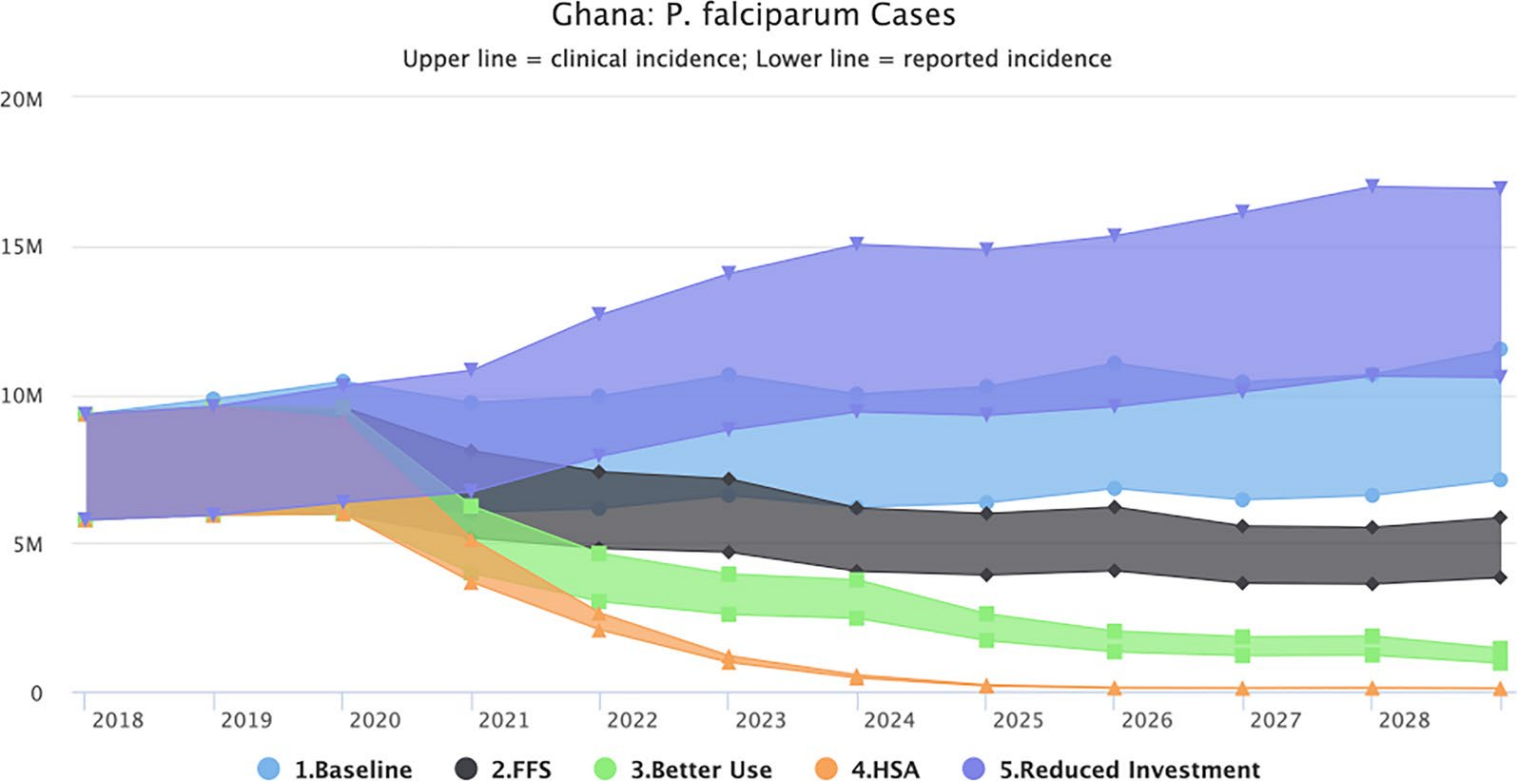
Reverse scenario 1 (predicted impact of removing IRS, SMC and reducing LLIN coverage by 50%)

Removing IRS, SMC and reducing LLIN coverage by 50% will result in an almost immediate upsurge of cases. By 2028, reported cases increase to more than 10.6 million (Fig. 5).

#### Reverse scenario 2

**Fig. 6:**
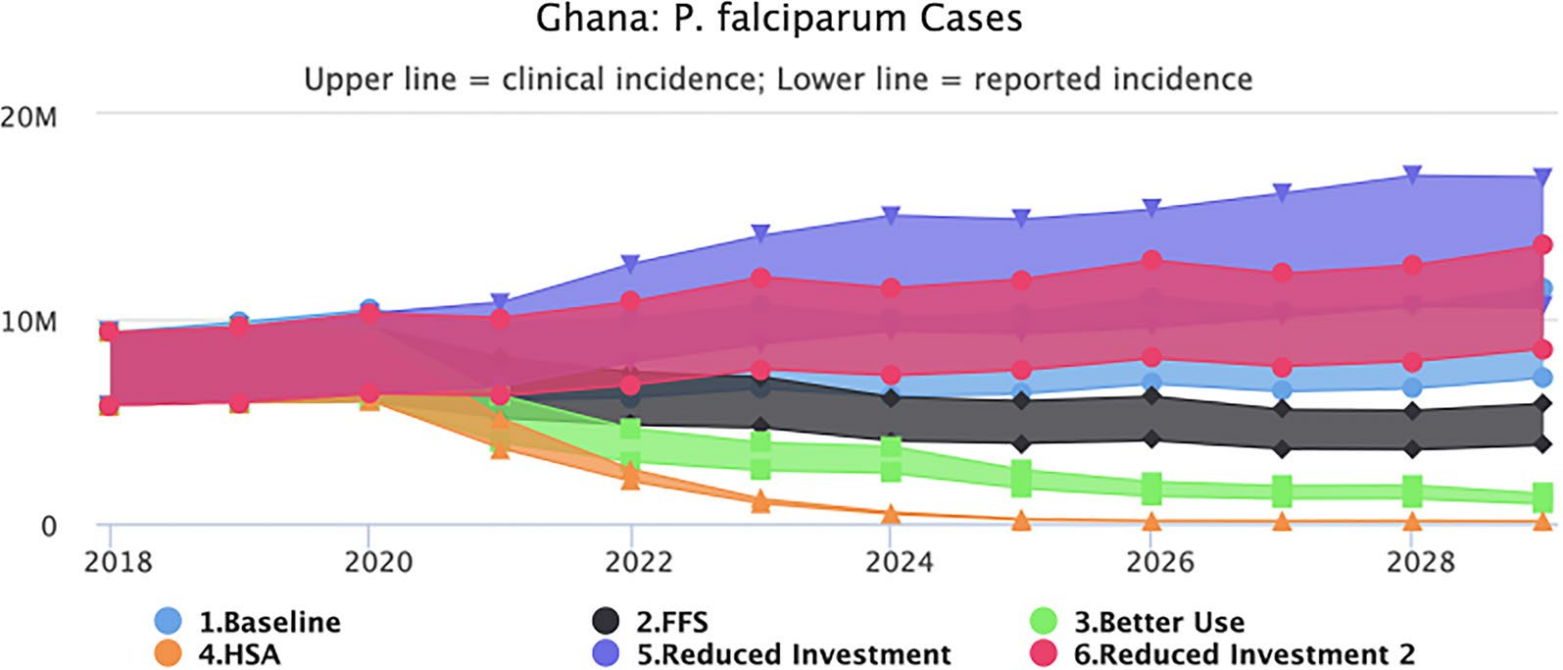
Reverse scenario 2 (predicted impact of removing IRS and SMC)

Removing IRS and SMC similarly reverse the gains made with reported cases rising to over 8.5 million by 2029 and clinical cases to 13.5 million (Fig. 6).

#### Zonal differences

**Fig. 7:**
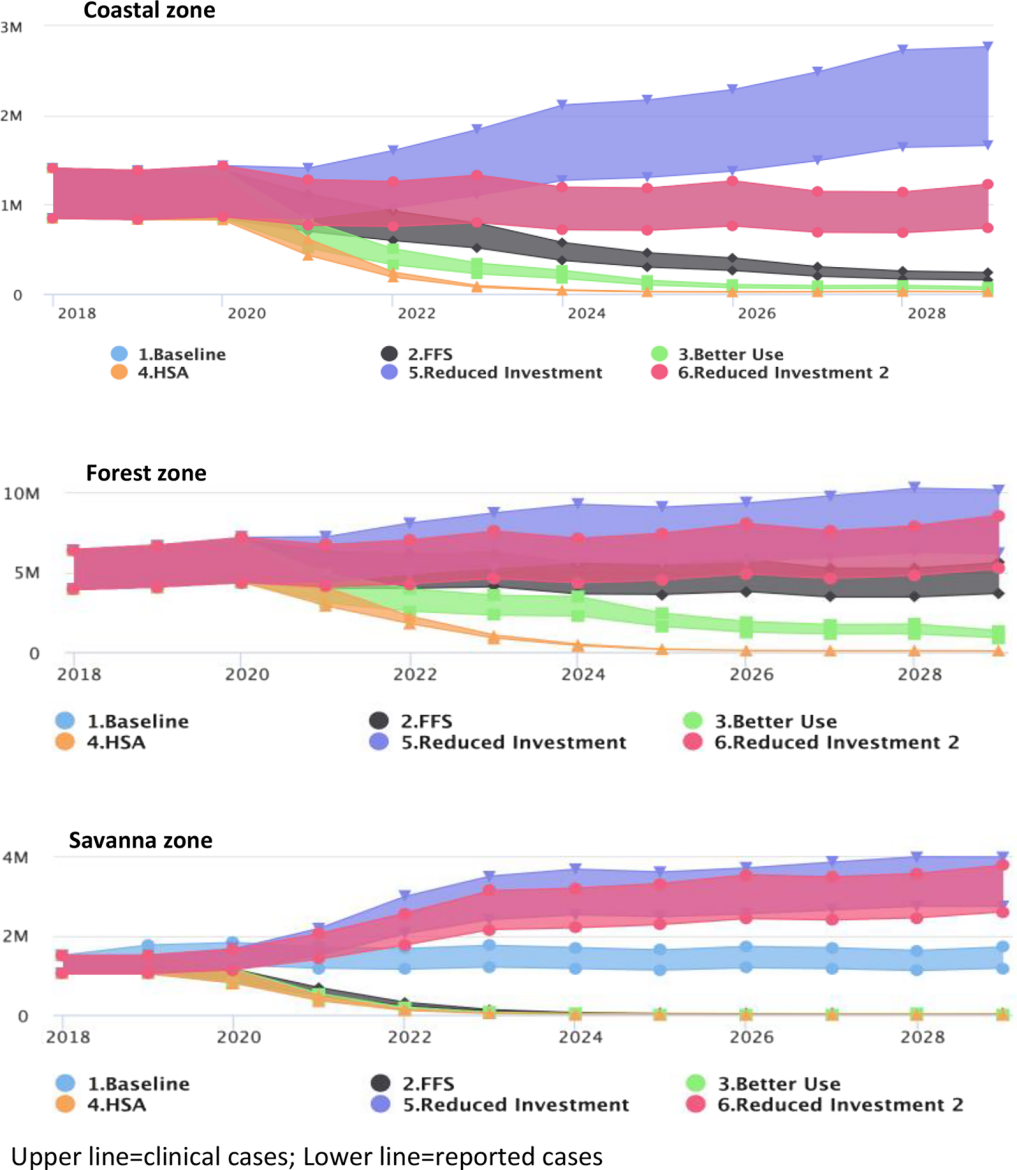
Predicted *P. falciparum* cases by zone for all modelled scenarios. Predictions of impact of interventions vary by zone (Upper line = clinical cases; Lower line = reported cases)

Figure 7 illustrates the zonal differences of intervention impact. Better net use and the fully funded scenario have less of an impact on transmission in the forest zone compared to the other two zones. Indeed, it would appear that this zone, having the largest case load, has the greatest impact on the total national response and hence experiences the greatest impact from the HSA scenario.

**Fig. 8:**
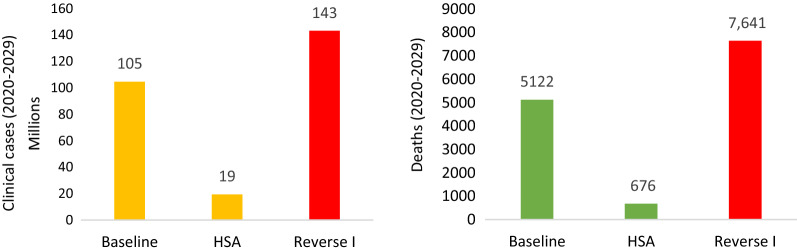
Predicted number clinical cases and deaths 2000–2029 for the baseline, HSA and reverse scenarios (removing IRS, SMC and reducing LLIN coverage by 50%)

Figure 8 summarizes the total clinical cases and deaths (2020–2029) with the baseline, HSA and reverse scenarios. Over the course of 10 years, the baseline scenario will result in 105 million clinical cases and 5122 deaths. The accelerated health system scenario will result in 19 million cases and 676 deaths while the reverse I scenario will result in 143 million cases and 7642 deaths.

### Cost projections

**Fig. 9:**
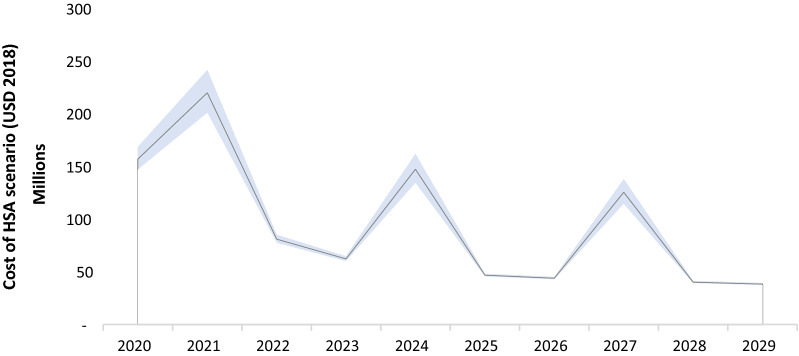
Cost projections for the HSA (elimination) scenario 2018–2030 (median, ± 25%). Elimination is predicted to cost a total of USD 961.3 million over 10 years (2020–2029) with the HSA scenario

The cost of implementing the HSA scenario is depicted in Fig. 9. The model predicted that it will cost a total of USD 961.3 million over 10 years (2020–2029) to implement the HSA scenario (to reach elimination). This equates to about USD 133 million annually for the first 5 years, assuming that the aggressive interventions predicted by the model are implemented in the most efficient way. This includes the integrated health system cost of treating outpatients and inpatients. The cost for the HSA scenario in 2020, not taking integrated costs into account, is USD 41.6 million. The peaks represent LLIN procurement for national mass campaigns which occur every 3 years in Ghana. To account for potential underestimation of reported cases, both reported and estimated cases were used to calculate modelled costs and benefits.

### Benefits estimation

The HSA scenario will avert 85.6 million clinical cases, 51.3 million reported cases and 4468 deaths. Economic benefits of USD 31.73 billion (NPV) are generated through reductions in deaths, cases, and household and healthcare system spending as well as increases in productivity at a cost of USD 961.34 million (2020–2029).

Reducing funding for IRS and SMC will incur additional (to the current baseline scenario) economic losses of USD 4.4 billion in addition to an additional 13 million clinical cases, 8.9 million reported cases and 1350 deaths. Further reducing funding for LLINs by 50% as well will incur economic losses 14.1 billion in addition to an additional 38.2 million clinical cases, 24.4 million reported cases and 2497 deaths. A summary of the results of various scenarios is shown in Table 3.

**Table 3:** Median costs and benefits of the baseline response against malaria compared to counterfactuals, 2020–2030

Scenario comparisons (Baseline—Intervention)	Clinical cases averted	Reported cases averted	Deaths averted	Economic benefits (NPV USD)	Cost (USD) (discounted)
Fully funded NSP scenario vs baseline	37,434,462	21,221,433	2683	14.1 billion	1.31 billion
Elimination scenario (HSA) vs baseline	85,571,086	51,251,099	4468	31.7 billion	961.3 million

	Additional clinical cases	Additional reported cases	Additional deaths	Additional economic losses (NPV USD)	
Baseline vs reverse I	38,220,597	24,411,310	2497	14.1 billion	
Baseline vs reverse II	12,974,304	8,914,008	1350	4.4 billion	

### Return on investment

Implementing the HSA scenario which will allow the country to progress to elimination and will produce a return on investment of 32:1 between 2020 and 2029 (Table 3).

### Gaps in malaria financing

The expected influx of financing (both domestic and donor) was compared with the projected cost of the malaria programme from 2020 to 2021. The modelled cost of the elimination scenario was estimated at 961.3 million (2020–2029). This equates to about USD 133 million annually for the first 5 years, assuming that the aggressive interventions predicted by the model are implemented in the most efficient way. This includes the integrated health system cost of treating outpatients and inpatients.

In addition to domestic government financing, Ghana received about USD 44 million from the Global Fund in 2018 and an USD 29 million from other donors [5]. In order to scale up the interventions needed, the remaining USD 60 million annually will need to be met by domestically. It is important to note that these figures do not account for the increased levels of co-financing levels that may be required by the Global Fund.

## Discussion

The current NSP was developed as a “malaria control” strategy rather than an “elimination” strategy. Therefore, as expected, implementing the interventions outlined therein will improve malaria control in Ghana and lower mortality and morbidity, but will not significantly lower transmission rates to levels that allow the programme to embark on malaria elimination. Elimination will only be achieved through the increased use of nets, improved health-seeking behaviour and strengthened malaria case management at the facility level as modelled in the HSA scenario.

The findings also indicate that the effects of the interventions vary by epidemiology and geographical zone. In the Forest Zone, which has the highest number of cases, better case management and increased treatment-seeking has the biggest impact in lowering transmission. In contrast, the Coastal and Savanna Zones the fully funded scenario and better net use and have more of an impact. The Savanna Zone has the lowest number of total cases and the fully funded scenario (NSP interventions) predicted a reduction in transmission significantly. These findings indicate that as transmission declines, there is a need to better stratify the risk of transmission in the various areas and target interventions purposefully to make an impact.

This analysis found that the cost of implementing the most effective scenario was USD 961.3 million over 10 years (2020–2029) equating to about USD 133 million annually for the first 5 years, assuming interventions are implemented efficiently. The health and economic benefits are enormous. Reducing transmission to elimination levels will avert 85.6 million clinical cases, 51.3 million reported cases and 4468 deaths. Economic benefits of USD 31.73 billion are generated through reductions in deaths, cases, and household and healthcare system spending as well as increases in productivity. The economic return is significant at 32 times the investment. This by far exceeds the threshold on returns that are considered to be high impact investments. In contrast, reducing funding for IRS and SMC will incur additional economic losses of USD 4.4 billion, an excess of 13 million clinical cases, 8.9 million reported cases and 1350 deaths. Further reducing funding for LLINs by 50% will incur economic losses USD 14.1 billion in addition to an excess of 38.2 million clinical cases, 24.4 million reported cases and 2497 deaths.

The benefits of investing in malaria elimination are likely to be undervalued as they exclude certain macro-economic costs that extend far beyond the health system. Studies have shown that indirect costs of malaria account for a large share of societal costs due to its debilitating effect on the economy through employee and school absenteeism, cognitive development in children as well as macroeconomic development by limiting foreign investments and tourism [26–30]. These have not been included due to the lack of accurate data to quantify these effects and to directly attribute them to malaria. Other costs to the health system such as cost of drug and insecticide resistance, the cost of higher price alternatives, the cost associated with their implementation, and the cost of research and development have also been omitted. The total income approach was used to compute income losses from malaria mortality. Although this methodology provides more generous estimates of economic losses than other methods, it is unlikely to account for all the aforementioned losses.

There are several limitations to the data and methods used in this study. Obtaining accurate data on the cost of programme operations, particularly in an integrated health system, is challenging. Several malaria programme resources are shared across other public health programmes, particularly for activities that are financed through government funding. Facility staff perform other health functions and therefore outpatient and inpatient costs are derived from estimates from the NHA.

The scenarios were developed in collaboration with the NMCP based on the current knowledge of interventions and strategic direction of the programme. As the programme progresses and transmission is reduced, these interventions are likely to the reviewed and revised.

Despite the robust benefits associated with investing in malaria, Ghana’s programme is likely to face a gap in funding in the immediate future. Funding for malaria from government sources met less than 30% of the total needs. Ghana is currently highly dependent on financing from the Global Fund at USD 36 million per year on average [31]. The current Global Fund grant ends in 2020. A funding request to the Global Fund for malaria will be developed for the period of 2021–2023. However, given that Ghana is already a lower middle-income country, as cases decline, it is unlikely that the Global Fund will maintain its current levels of funding, particularly if the co-financing requirement remains unpaid. These financing gaps will need to be met by increased domestic financing. A robust resource mobilization strategy bolstered by the epidemiological and economic evidence generated by this report will be needed.

In 2015, a *Resource Mobilization Plan for National Malaria Control Strategy (2014*–*2020)* [32] was developed and laid the foundation for resource mobilization efforts for malaria in Ghana. The subsequent *Ghana Health Service Resource Mobilization Strategy for National Malaria Control and Elimination (2019*–*2023)* [4] is awaiting finalization and ratification. At the same time, several actions have since been taken: The Ghana Malaria Foundation (GMF), established in 2015/2016 and officially inaugurated in 2017 has been accredited as a limited guarantee corporate body. However, a renewed effort incorporating the evidence from this report is needed, which includes outlining clear actions for implementing identified strategies for resource mobilization.

Ghana’s private sector is large and growing, and its engagement in supporting the implementation of malaria control interventions during the past decade has increased. Engagement includes corporate social responsibility programmes (e.g., through oil companies), workplace wellness programmes (e.g., plantations), marketing of effective malaria treatment and prevention products and services and other public–private partnerships including market catalyzation for malaria control products. Several opportunities exist to expand these efforts in support of the new strategy.

Remittances are one of the largest sources of funding flows next to international aid. Providing a small portion of each transaction to malaria — possibly through a Corporate Social Responsibility model via mobile transaction companies should be explored, especially since these funds could be applied to a matching fund scheme. In 2017, remittances made up 5.1% of Ghana’s GDP to the tune of USD 2.19 billion and which, according to the Bank of Ghana, grew to USD 3.52 billion in 2018 [32].

Public and private sector donors can enter into matching fund programmes, which would substantially increase donations for Ghana’s national malaria programming. For example, in Mozambique, DFID and the Global Fund matched Ecobank Foundation’s USD 750,000 donation for an LLIN campaign in the country, raising the value of that donation to USD 2.5 million. Ghana could explore developing a matching co-financing facility with private sector companies, such as those from the extraction industry and financial sector, and other donors. Other opportunities to create an enabling environment include tax incentives from the government for private sector involvement.

Engagement with companies to raise funds from customers and employees to contribute to the GMF may be a viable option. Potential mechanisms are voluntary contributions through online banking platforms or similar online systems. For example, the Ecobank Foundation invited Ecobank employees on World Malaria Day 2017 to donate USD 3.00 for an LLIN to be distributed to vulnerable populations. With little effort, out of 16,000 employees, Ecobank raised USD 22,000. This can serve as an example for other companies to help fill the NMCP gaps [32].

Currently, the Government collects taxes on tobacco products, alcohol and soda, tourism and airport levies, petroleum revenues, lottery funds and others. However, none of these are currently earmarked for health and present a potential opportunity for increased resources. By law, 0.5% of new funding allocated within the District Assembly Common Fund is mandated to be used toward malaria control and elimination efforts at the district level. These funds are frequently not being used for this purpose and advocacy to ring-fence the funds is needed.

Other opportunities include using existing resources more efficiently. The current cost of the programme does not include measures to improve efficiencies. Targeted interventions such as vector control to high risk areas and populations will likely provide considerable cost-efficiencies. The implementation of efficiency measures in malaria programming can also serve as an advocacy tool to approach existing and new donors. These strategies can be made more effective through the use of key influencers or ambassadors to ensure accountability.

## Conclusions

The findings indicate that while the interventions outlined in the NSP will lower transmission of malaria, they are not likely to allow the elimination of the disease. Elimination will only be achieved through increased use of nets, improved health-seeking behaviour and strengthened malaria case management at the facility level. The effects of these interventions vary by zone and there is a need for risk stratification and to target interventions particularly as transmission declines and the country moves closer to elimination. Furthermore, as cases decline and the epidemiology of the disease changes, a similar exercise with revised elimination interventions will need to be conducted with the additional costs of surveillance built in.

Eliminating malaria will avert 85.6 million cases and 4500 deaths. 1.06 billion days of employee absenteeism will be avoided conferring economic benefits of 31.73 billion providing a return on investment of 32:1. Reducing investments and a resulting resurgence will lead to an additional 38.2 clinical cases, 2500 deaths and additional economic losses of USD 14.1 billion. There are several critical reasons why malaria should receive a special focus for financing. Malaria is a major ongoing cost driver burdening national health systems and eliminating the disease will confer public health benefits as well as major cost savings to national health systems. Although the short-term investment needed may seem substantial at USD 961.3 million over 10 years (2020–2029), front loading investments will provide cost savings in the longer term as well as substantial health and economic returns. At the same time, Ghana could be at the frontier of elimination in Africa amongst the identified WHO High-Burden High-Impact countries in Africa. There is a need for a robust and effective resource mobilization and advocacy strategy backed by the evidence produced by this body of work.

## Supplementary information

**Additional file 1.** Inputs and assumptions used in the analysis.

## Data Availability

The datasets used and/or analysed during the current study are available in a separate database [11] online and as additional tables included in this submission. Other raw data is available from the corresponding author on reasonable request.

## Abbreviations

ACT: Artemisinin-based combination therapy
AL: Artemether–lumefantrine
BCR: Benefit-cost ratio
CCP: Center for Communication Programs
CHPs: Community Health Planning Services
CHW: Community Health Workers
DFID: Department for International Development
ERC: Ethical Review Committee
GDP: Gross Domestic Product
GHC: Ghanaian Cedi
GHS: Ghana Health Service
Global Fund: Global Fund to Fight AIDS, Tuberculosis and Malaria
GNI: Gross national income
GOG: Government of Ghana
GVA: Gross value added
IEC: Information, education, and communication
IPT: Intermittent preventive treatment for pregnant women
IRS: Indoor residual spraying
ITN: Insecticide-treated mosquito net
JHU: Johns Hopkins University
LLIN: Long-lasting insecticide-treated mosquito net
MICS: Multiple indicator cluster survey
MIS: Malaria indicator survey
MOP: Malaria operational plan
MOH: Ministry of Health
NHIS: National Health Insurance Scheme
NMCP: National Malaria Control Programme
NPV: Net present value
NSP: National malaria strategic plan
PMI: President’s malaria initiative
RDT: Rapid diagnostic test
ROI: Return on investment
SBC: Social behavioural change
SMC: Seasonal malaria chemoprevention
SP/AQ: Sulfadoxine-pyrimethamine and amodiaquine
SP: Sulfadoxine-pyrimethamine
USAID: United States Agency for International Development
USD: United States Dollar
WHO: World Health Organization
