# Supplementary material for: Estimating the risk of declining funding for malaria in Ghana: the case for continued investment in the malaria response
Source: Malar J. 2020 Jun 1;19:196. doi: 10.1186/s12936-020-03267-9 (PMC7268595; doi:10.1186/s12936-020-03267-9)
Supplement: Supplementary file 1 — Additional file 1. Inputs and assumptions used in the analysis. [file 12936_2020_3267_MOESM1_ESM.docx]

**Additional Table S1. Inputs and assumptions used in the analysis**

|  | **Value (USD)** | **Source** |
| --- | --- | --- |
| Cost of OP malaria treatment (without medicines) | 14.00 | NHA [22,23] |
| Cost of IP malaria treatment per day (without medicines) | 110 (5 days) | NHA [22,23] |
| Cost of RDT per case diagnosed | 1.00 | NMCP |
| Cost of *P. falciparum* medicines per OP case | 1.86 | NMCP |
| Cost of *P. falciparum* medicines per IP case | 29.86 | NMCP |
| Cost per person protected with LLIN (campaign) | 5.32 | NMCP |
| Cost per person protected with LLIN (routine) | 7.13 | NMCP |
| Cost of IRS per household protected | 5.43 | NMCP |
| Cost of SMC per patient protected | 0.92 | NMCP |
| Annual cost of training, supervision and monitoring per capita | 0.17 | [24 7,9]^a^ |
| Annual cost of SBC per capita (treatment) | 0.06 | [24,7,9]^a^ |
| Annual cost of SBC per capita (LLIN) | 0.10 | [17] |
| OOP per OP malaria case | 4.91 | [25] |
| OOP per IP malaria case | 24.55 | ^b^ |
| Economics | | |
| GDP per capita (USD) | 1807.10 | [3] |
| Coefficient for VLY calculation | 4.20 | [13] |
| Discount rate (%) | 3.00 |  |
| Exchange rate (2018 mid-year) | 5.78 | [10] |
| Mortality | | |
| Life expectancy at 40 years | 33.20 | [14] |
| Life expectancy at 2.5 years | 66.83 | [14] |
| Epidemiology and length of disease | | |
| Proportion of malaria cases that are treated OP | 93% | Model |
| Proportion of malaria cases that are treated IP | 7% | Model |
| Proportion of severe malaria cases in adults |  | ^a^ |
| Proportion of severe malaria cases in children < 5 years |  | ^a^ |
| Length of OP malaria case (days lost) | 5.85 | [26] |
| Length of IP malaria case (days) | 10.79 | [26] |
| Length of IP malaria hospitalization (days lost) | 5.00 | NMCP |

^a^ Calculated by authors using data from the references cited. ^b^ Assumption made by authors

**Other assumptions**

1. Annual cost of SBC per capita (treatment) deduced as 70% of the PMI investments for training, supervision and monitoring and supply chain management support (2016-18).
2. OOP expenditures for IP = 5 x OOP for OP (5 days in hospital)
